# Identification of Factors Determining Patterns of Serum C-Reactive Protein Level Reduction in Response to Treatment Initiation in Patients with Drug-Susceptible Pulmonary Tuberculosis

**DOI:** 10.3390/antibiotics13121216

**Published:** 2024-12-14

**Authors:** Agnija Kivrane, Viktorija Ulanova, Solveiga Grinberga, Eduards Sevostjanovs, Anda Viksna, Iveta Ozere, Ineta Bogdanova, Ilze Simanovica, Inga Norvaisa, Leonora Pahirko, Dace Bandere, Renate Ranka

**Affiliations:** 1 Pharmacogenetic and Precision Medicine Laboratory, Pharmaceutical Education and Research Centre, Riga Stradins University, Konsula Street 21, LV1007 Riga, Latvia; 2 Latvian Biomedical Research and Study Centre, Ratsupites Street 1, k-1, LV1067 Riga, Latvia; 3 Laboratory of Physical Organic Chemistry, Latvian Institute of Organic Synthesis, Aizkraukles Street 21, LV1006 Riga, Latvia; 4 Department of Infectiology, Riga Stradins University, Dzirciema Street 16, LV1007 Riga, Latvia; 5 Centre of Tuberculosis and Lung Diseases, Riga East University Hospital, Stopini Region, LV2118 Upeslejas, Latvia; 6 Faculty of Science and Technology, University of Latvia, Jelgavas Street 3, LV1004 Riga, Latvia; 7 Department of Pharmaceutical Chemistry, Pharmaceutical Education and Research Centre, Riga Stradins University, Konsula Street 21, LV1007 Riga, Latvia

**Keywords:** tuberculosis, pharmacokinetics, anti-tuberculosis drugs, C-reactive protein, treatment response

## Abstract

**Background:** Serum C-reactive protein (CRP) levels vary depending on radiological and bacteriological findings at the time of tuberculosis (TB) diagnosis. However, the utility of this biomarker in monitoring response to anti-TB treatment and identifying patients at risk of treatment failure is not well established. **Objectives:** This study evaluated the impact of patients’ baseline characteristics and anti-TB drug plasma exposure on the early reduction in serum CRP levels and its relationship with treatment response. **Methods:** We enrolled 42 patients with drug-susceptible pulmonary TB, who received a standard six-month regimen. The plasma concentrations of four anti-TB drugs were analysed using LC-MS/MS. Clinically relevant data, including serum CRP levels before and 10–12 days after treatment initiation (CRP_10–12d_), were obtained from electronic medical records and patient questionnaires. **Results:** In 10–12 days, the median serum CRP level decreased from 21.9 to 6.4 mg/L. Lower body mass index, positive sputum-smear microscopy results, and lung cavitations at diagnosis were related to higher biomarker levels at both time points; smoking had a more pronounced effect on serum CRP_10–12d_ levels. Variability in anti-TB drug plasma exposure did not significantly affect the reduction in serum CRP levels. The serum CRP_10–12d_ levels, or fold change from the baseline, did not predict the time to sputum culture conversion. **Conclusions:** Disease severity and patient characteristics may influence the pattern of early CRP reduction, while anti-TB drug plasma exposure had no significant effect at this stage. These early changes in serum CRP levels were not a predictor of response to anti-TB therapy.

## 1. Introduction

With a global incidence of 10–12 million cases per year, tuberculosis (TB) remains an alarming public health problem, even in the era of advanced medical technologies [1]. Despite the potential benefits of the four-month rifapentine-based regimen recently introduced in the World Health Organization (WHO) guidelines on the treatment of drug-susceptible tuberculosis (DS-TB), issues with rifapentine availability prevent the incorporation of this regimen into national TB programmes worldwide [2,3,4]. Therefore, the standard of care for DS-TB in most countries within the WHO European Region, including Latvia, remains the six-month regimen consisting of rifampicin (RIF), pyrazinamide (PZA), ethambutol (ETB), and isoniazid (INH).

In patients undergoing the lengthy anti-TB treatment course, monitoring of treatment response to identify those at risk of adverse treatment outcomes is the cornerstone of effective disease management. Once treatment is started, the alleviation of clinical symptoms, radiological improvement, and a reduction in the sputum bacillary load are generally expected after two to four weeks [5,6,7,8,9,10]. According to the WHO, the matter of concern is the patients who lack clinical response and fail to convert their sputum-smear and sputum culture by the end of the intensive phase of treatment, i.e., within the first two months of anti-TB drug administration [11]. Calderwood et al. [12] revealed that around 20% of patients fall into the category of delayed treatment response—an unfavourable short-term outcome indicating a limited bactericidal effect, which may translate into treatment failure or relapse.

C-reactive protein (CRP), an acute-phase protein, is widely employed to assess and monitor inflammation in acute and chronic conditions [13]. Mechanistically, inflammatory stimuli resulting from exposure to infectious agents or tissue damage activate the macrophages, which then release proinflammatory cytokines, namely, IL-1, IL-6, and TNF-α, driving the expression of CRP, primarily in the hepatocytes [13]. In patients with TB, serum CRP levels were reported to correlate with disease severity and bacillary load at diagnosis [9,14,15,16,17,18,19]. Several studies exploring changes in serum CRP levels throughout the anti-TB treatment course have confirmed a substantial decline in the levels of this biomarker during the first weeks of treatment, reflecting the attenuation of the inflammation related to *Mycobacterium tuberculosis* (Mtb) clearance from the lungs and extrapulmonary sites [9,14,17,18,19,20]. Furthermore, Wilson et al. [20] showed that the failure to achieve a 55% reduction in serum CRP levels within the first two weeks of anti-treatment was a predictor of hospitalisation and death in patients with TB-HIV coinfection. Currently, point-of-care CRP testing is approved for screening of active TB in people living with HIV, but there is little role of this biomarker in monitoring treatment response due to inconclusive evidence regarding its association with bacteriological endpoints [14,17,18,19,21,22,23]. Considering the non-specific nature of CRP expression and the fact that its serum levels can considerably vary over the six-month treatment period, this biomarker could be more suitable for predicting short-term rather than long-term effects [13].

A variety of lifestyle-, health-, and disease-related factors bear the potential to slow down the progress of anti-TB therapy and, in some cases, endanger treatment success [21,23,24,25,26,27,28,29,30,31]. At the same time, pharmacokinetic/pharmacodynamic (PK/PD) studies have drawn attention to the variability in plasma concentrations of anti-TB drugs, along with considerable underexposure rates observed with recommended daily doses [23,32,33,34,35,36,37,38,39]. Maintaining PK/PD targets is highly important, especially given the complexity of eradicating Mtb [40]. Each of the four anti-TB drugs has a distinct mechanism of action and penetration rates into heterogenous tuberculous lesions, enabling them to target Mtb at different stages of replication [8,10,40,41,42,43]. In line with that, patients experiencing delayed treatment response and adverse treatment outcomes often exhibit lower plasma exposure (or related PK/PD indices) of one or more anti-TB drugs [23,33,34,35,36,37,39]. On the contrary, the role of anti-TB drug exposure in the reduction in TB-associated inflammation has not been extensively discussed; however, Prahl and colleagues [23] have described a negative correlation between serum CRP levels and INH plasma concentration during the intensive phase of treatment.

Thus, this study sought to (a) further investigate the influence of patients’ baseline characteristics and plasma exposure of four anti-TB drugs on the reduction in the serum CRP levels in response to treatment onset and (b) determine whether these patterns of serum CRP reduction are linked to the treatment response in Latvian patients with drug-susceptible pulmonary tuberculosis (DS-PTB).

## 2. Results

### 2.1. Baseline Characteristics of the Study Population

In total, 42 otherwise healthy patients with radiologically and bacteriologically confirmed DS-PTB were enrolled in the study; 81.0% (34/42) of the patients were males, and the mean age was 47 years (±14 years). A detailed patient demographic and anthropometric structure, along with lifestyle and clinical characteristics, are provided in Table 1. At admission, all but five patients presented with clinical symptoms suggestive of TB, such as persistent cough, night sweats, and unexplained body weight loss. The baseline radiological assessment revealed that 73.8% (31/42) of the patients had tuberculous lesions in both lungs, and 61.9% (26/42) had cavitary disease. Also, two patients had developed pleuritis secondary to PTB. Nearly two-thirds of all investigated patients (27/42, 64.3%) had positive sputum-smear microscopy results at the baseline (Table 1). When characterising the degree of inflammation before starting anti-TB treatment, the median baseline serum CRP level (CRP_b_) in the study population was 21.9 mg/L (interquartile range [IQR]: 3.3–51.5 mg/L); significant differences were observed between patients with and without cavitary lesions (median 36.3 mg/L [IQR: 19.8–66.6 mg/L] vs. 3.0 mg/L [IQR: 1.1–7.6 mg/L], *U* = 379, *p* < 0.001) and between patients with positive and negative sputum-smear microscopy results (median 29.2 mg/L [IQR: 8.2–60.6 mg/L] vs. 3.4 mg/L [IQR: 1.1–26.6 mg/L], *U* = 299, *p* = 0.011) (Table 2). In contrast, the localisation of lung lesions was not related to the levels of this biomarker (*U* = 203, *p* = 0.365). The levels of serum CRP_b_ inversely correlated with body mass index (BMI) (Spearman’s rho = −0.39, *p* = 0.011) and varied across BMI categories (*H*_(2)_ = 6.46, *p* = 0.040). After correction for multiple comparisons, a difference of marginal significance was found between underweight and overweight patients (adjusted *p* = 0.050). Concerning other baseline characteristics, serum CRP_b_ levels did not vary depending on the patient’s biological sex, age, or smoking status.

### 2.2. Assessment of Anti-TB Drug Plasma Exposure

The data on the resulting plasma exposure of the four anti-TB drugs after single-dose administration are summarised in Table 3. In conformity with the criteria proposed by Alsultan et al. [45], the peak plasma concentration (C_max_) evaluated 2 h post-dose was below the therapeutic range for RIF and INH (median 1.9 µg/mL [IQR: 0.3–5.5 µg/mL] vs. 8.0 µg/mL and mean 2.6 µg/mL [±1.38 µg/mL] vs. 3.0 µg/mL, respectively), whereas for the remaining drugs, it was within the target range. As such, the highest underexposure rate of 92.9% (underexposed 39 out of 42 patients) was detected for RIF, followed by INH, ETB, and PZA, with insufficient plasma concentration accordingly in 54.8% (23/42), 26.2% (11/42), and 9.5% (4/42) of patients. The prevalence of simultaneous four-drug underexposure was 7.1%.

### 2.3. The Reduction in Serum CRP Levels in Response to Anti-TB Treatment and the Impact of Anti-TB Drug Plasma Exposure

**Figure 1:**
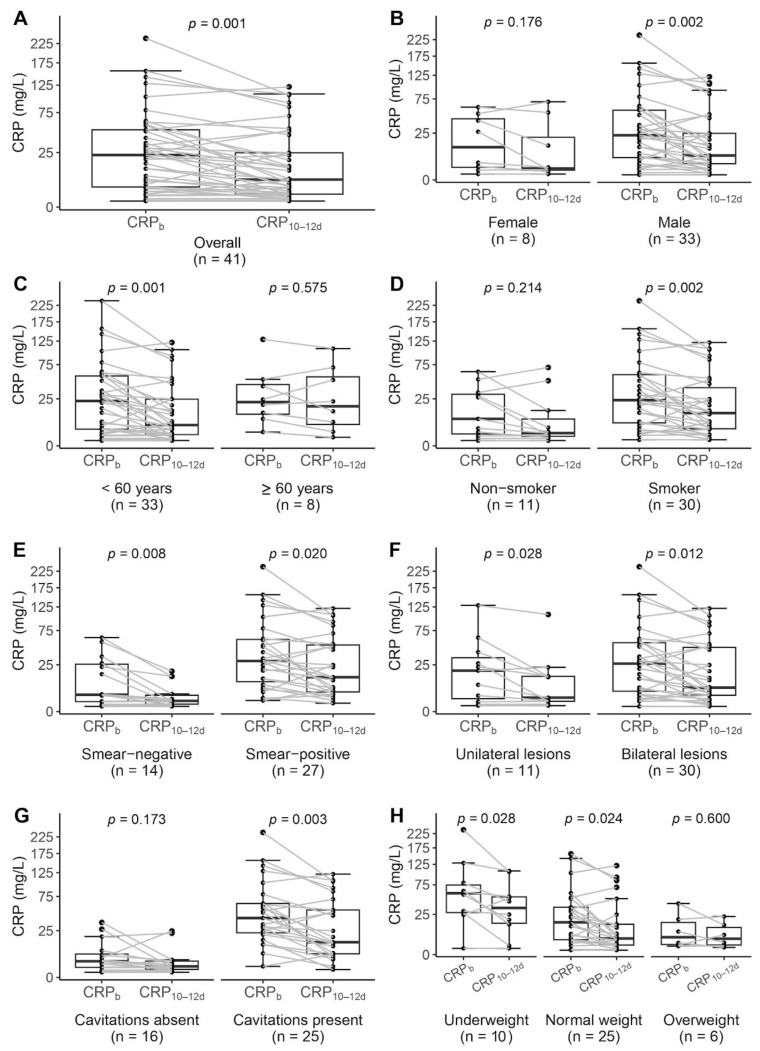
Changes in serum CRP levels measured before anti-tuberculosis treatment initiation (CRP_b_) and 10–12 days afterwards (CRP_10–12d_) (**A**) and comparison across different subcategories ((**B**)—biological sex; (**C**)—age; (**D**)—smoking status; (**E**)—baseline sputum-smear microscopy results; (**F**)—localisation of lung lesions; (**G**)—presence of cavitary lesions; (**H**)—BMI category) using the Wilcoxon signed-rank test. BMI categories were assigned according to WHO classification [44]: underweight—BMI < 18.5 kg/m^2^; normal weight—18.5 kg/m^2^ ≤ BMI < 25.0 kg/m^2^; overweight—BMI ≥ 25.0 kg/m^2^. The upper and lower margins of the boxes indicate the first and third quartiles, respectively, with the horizontal line within the box indicating the median. The whiskers show the highest and lowest values within 1.5 times the interquartile range from the first and third quartile. The grey lines connect the paired data points for each patient. A *p* value of <0.05 was considered statistically significant. Abbreviations: CRP—C-reactive protein.

Overall, in the study population, the median serum CRP level after 10–12 days of anti-TB treatment (CRP_10–12d_) decreased to 6.4 mg/L (IQR: 1.4–34.2 mg/L, *W* = 142, *p* = 0.001), thereby meeting the reference range of <8 mg/L established by the clinical laboratory of Centre of Tuberculosis and Lung Diseases at Riga East University Hospital. Even in the subcategories with median serum CRP_10–12d_ levels above this threshold, except in patients aged ≥60 years, the decline was significant (Figure 1 and Table 2). As seen with CRP_b_, serum CRP_10–12d_ levels varied depending on the baseline sputum-smear microscopy results (*U* = 324, *p* < 0.001) and the presence of cavitary lesions at the radiological examinations (*U* = 356, *p* < 0.001), and correlated with BMI (Spearman’s rho = −0.33, *p* = 0.035) (Table 2). It is noteworthy that the difference in the median level of this biomarker between smokers and non-smokers was more pronounced than with CRP_b_ and reached significance (median 12.1 mg/L [IQR: 3.2–45.0 mg/L] vs. 1.8 mg/L [IQR: 0.8–14.1 mg/L], *U* = 233, *p* = 0.046). Despite the aforementioned variability in anti-TB drug exposure, the linear regression analysis did not demonstrate a significant relationship between serum CRP_10–12d_ levels and the PK parameters of the four anti-TB drugs (Table 3).

In the subgroup analyses, nearly one-third of patients (13/41; 31.7%) had serum CRP levels within the reference range at both time points (Group A). In 34.1% (14/41) of patients, serum CRP levels decreased ≥2 times from the baseline or reached the reference range (Group B), while the same proportion of patients did not meet this criterion (Group C). Referring to Supplementary Table S1, the baseline characteristics were consistent across the groups, except for radiological and bacteriological findings. After applying Bonferroni correction, it was observed that patients in Group A less frequently presented with cavitary disease compared to those in Group B (adjusted *p* = 0.003) and Group C (adjusted *p* < 0.001), as well as with positive sputum-smear microscopy results compared to Group C (adjusted *p* = 0.012). The estimated marginal means of PK parameters slightly varied across the three groups, but statistical significance was not achieved (Supplementary Table S2). Further analysis to assess the effect of previously discussed factors on the odds of reaching at least a 2-fold reduction in serum CRP levels was limited by statistical power.

### 2.4. Treatment Response and Its Relationship with Early Changes in Serum CRP Levels

Thirty-eight patients (90.5%) fully recovered from the Mtb infection and met the WHO definition for cured [11]; three patients completed the six-month anti-TB course, but their treatment outcome could not be classified as cured (7.1%); one patient was lost to follow-up (2.4%). In patients whose treatment outcome was “cured” (n = 38), the median time to sputum culture conversion (tSCC) was 56 days (IQR: 27–79 days), and 55.3% (21/38) had their sputum culture converted within the first two months, while at the end of the sixth month, the culture conversion rate reached 97.4% (37/38). Although data distributions were overlapping, patients with delayed treatment response (tSCC ≥ 60 days) tended to have higher serum CRP_10–12d_ levels compared to those with tSCC < 60 days (median 13.0 mg/L [IQR: 3.1–66.9 mg/L] vs. median 5.4 mg/L [IQR: 1.5–20.4 mg/L], *U* = 211, *p* = 0.220).

According to the subgroup analysis, the median tSCC in Groups A and B—representing patients with serum CRP levels within the reference range at both time points, and those who achieved at least a 2-fold reduction in the levels of this biomarker (or reached the reference range) after 10–12 days of therapy—was 46 days (IQR: 27–77 days for Group A and IQR: 25–73 days for Group B, respectively). Meanwhile, in Group C, which included patients who did not fulfil the criteria for CRP reduction, the median tSCC was 66 days (IQR: 36–86 days). The difference in tSCC between all three groups was insignificant (*H*_(2)_ = 0.63, *p* = 0.626).

In the univariate Cox regression models, neither serum CRP_10–12d_ levels nor the subgroups assigned based on changes in serum CRP levels over the specified period were significant predictors for tSCC (Supplementary Table S3). The best-performing multivariate models, which include clinically relevant factors commonly associated with delayed treatment response, are given in Table 4. However, including these covariates did not improve the predictive value of the investigated CRP parameters.

## 3. Discussion

At first, we conducted a series of correlation analyses and group comparisons to explore variations in serum CRP levels based on patient characteristics. Among the factors naturally contributing to the variability in the serum CRP levels, we observed differences in the median values when analysing the impact of biological sex, age, and smoking status, though these differences did not reach significance [46,47,48,49,50,51]. Only BMI showed a moderately strong negative correlation with serum CRP_b_ levels. The difference between underweight patients and those categorised as overweight was of marginal significance, yet the levels of this biomarker differed 10-fold between these BMI subcategories. While serum CRP levels typically increase with BMI in healthy adults, the opposite trend is reported in patients with TB [52,53,54]. In this context, malnutrition is a well-recognised risk factor for developing TB, whereas wasting is a common symptom associated with the advanced forms of the disease, delayed treatment response, and adverse treatment outcomes [27,29,52,53,54,55,56,57].

Relating to characteristics specific to TB, several studies have shown that serum CRP levels complement radiological and bacteriological findings obtained at the time of diagnosis [9,14,15,16,17,18,19]. Similarly, our patients with radiologically confirmed cavitary disease and positive sputum-smear microscopy results had higher serum CRP_b_ levels than their counterparts. We did not observe the impact of the extent of lung involvement; meanwhile, other studies report conflicting findings [14,15,18,58,59]. The discrepancy likely arises from the differences in the type of radiological examination and methodology utilised to estimate the TB-affected lung area.

In 10–12 days, the median serum CRP level in our study population decreased from 21.9 to 6.4 mg/L, falling within the reference range (<8 mg/L). This complies with the results from the studies investigating CRP kinetics in patients with TB, where a drop in the levels of this biomarker occurred between the first and fifth weeks of treatment [14,18,19,20]. Subsequent analyses revealed that underweight patients, smokers, those with lung cavitations, and positive sputum microscopy results before anti-TB treatment onset experienced a significant decrease in serum CRP levels compared to the baseline, but their serum CRP_10–12d_ levels were still distinct from the target. As described elsewhere, low body weight and smoking have additive effects on inflammation and serum CRP levels in patients with TB, but the highlighted radiological and bacteriological findings, characteristic of the advanced disease, are independently associated with high-degree inflammation [14,15,16,17,18,49,50,51,53,54]. In addition, these factors determined slower sputum bacillary clearance in the studies by Nijenbandring de Boer et al., Kanda et al., and other groups [24,25,27,28,29]. It is, therefore, not surprising that patients with a combination of these factors may require a longer time to sterilise the infection and reach the established serum CRP reference range. Even though, in our work, patient age did not significantly correlate with the serum CRP levels at any time point, possibly due to the high proportion of patients aged <60 years (81%), the impact of age manifested as the inability of patients aged 60 years and older to achieve a significant decrease in the level of this biomarker within the specified period. Ageing is a degenerative process with effects extending to the immune system (immunosenescence) and inflammation (inflammaging) [60]. Hence, the slow decline in serum CRP levels, at least in part, could be attributed to intensified and prolonged proinflammatory cytokine production, e.g., TNF-α, IL-6, and IL-1β, as well as to the altered function of immune cells, including macrophages and CD4+ T lymphocytes—the crucial components of immune defence against Mtb [60,61].

Given the observed variability in the reduction in serum CRP levels and the absence of clinical recommendations on expected reduction patterns or timeframes, we applied the approach described by Wilson et al. [20], with minor modifications, to categorise changes in the levels of this biomarker for subgroup analyses.

After stratifying patients based on a 2-fold reduction in serum CRP levels from the baseline and attainment of the reference range after 10–12 days of anti-TB therapy, it was found that 31.4% did not achieve the expected decrease (Group C). They more frequently exhibited lung cavitations or positive sputum-smear microscopy results at the initial clinical evaluation compared to patients with serum CRP levels within the reference range at both time points (Group A) and those who achieved the stated goal (Group B). These observations align with the results obtained in our primary analyses and reaffirm the role of disease severity in the early reduction in inflammation.

Next, we investigated whether anti-TB drug plasma exposure accounts for the variability in serum CRP levels on the 10th–12th day of treatment. The liquid chromatography–tandem mass spectrometry (LC-MS/MS) data showed suboptimal RIF plasma concentrations in 92.9% of patients at 2 h post-dose, while underexposure rates for other drugs ranged from 9.5% to 54.8%. Other studies have reported comparable rates and identified factors contributing to the observed underexposure [23,32,33,34,35,36,37]. From our study’s perspective, since the initiation of anti-TB treatment caused a significant decline in serum CRP levels, it appears that drug exposure at the site of infection was sufficient for effective early Mtb killing. Indeed, several groups of authors have documented the accumulation of anti-TB drugs in epithelial lining fluid and alveolar cells to varying extents, with plasma or serum concentrations being considerably lower or even below the therapeutic range [62,63,64]. Notably, a review published a few years ago underlined the discordance between widely used therapeutic ranges (suggested by Alsultan et al. [45]) and real-world data [65]. Nevertheless, an in-depth analysis of PK variability was beyond the scope of this study.

Compared to an earlier study [23], our results did not confirm a relationship between the PK parameters of any of the four anti-TB drugs and serum CRP_10–12d_ levels. In the subgroup analyses, patients whose CRP levels reduced <2 times (Group C) had similar anti-TB drug plasma exposure compared to those belonging to Groups A and B. Thus, in our setting, the variations in anti-TB drug plasma exposure did not result in clinically relevant consequences, although a long-term effect cannot be ruled out. It is plausible that differences in study design and patient characteristics may explain the conflicting results to some extent. In our study, the time between treatment onset and PK sampling was fixed, and the study population was homogeneous in terms of ethnicity, form of TB, comorbidities, care setting, and drug formulation used. Emphasising the significance of these discrepancies, PK/PD studies have reported that some of these factors may contribute to the PK variability of anti-TB drugs [37,66,67,68].

Finally, we evaluated serum CRP_10–12d_ levels and the patterns of early changes in serum CRP levels as independent predictors of tSCC. The median tSCC in our patients was 56 days, which could be considered a “good treatment response” [11]. We assume that the population structure and the absence of concomitant diseases determined the relatively fast sputum culture conversion, even in patients who did not achieve a rapid reduction in serum CRP levels after the initiation of anti-TB treatment (46 days in Group A and B vs. 66 days in Group C).

In general, our patients with delayed sputum culture conversion (tSCC ≥ 60 days) had higher, albeit not significantly, serum CRP levels 10–12 days after starting anti-TB treatment. Consequently, in Cox regression analysis, none of the studied factors predicted tSCC. Musteikienė et al. [18,21] recorded significantly higher baseline serum CRP levels for patients with SCC after Month 1 in both of their studies but also failed to observe the effect on sputum culture status in their later analyses. Djoba et al. [14] predicted sputum culture status by the end of the intensive phase of anti-TB treatment with more than 80% accuracy using a biomarker signature incorporating serum CRP levels at baseline and Week 1. Another study found that serum CRP levels were more strongly associated with sputum culture status at Week 8 vs. Week 12. Yet, employing biomarker combinations did not improve the predictive performance [17]. Interestingly, the relationship between serum CRP levels and treatment response in the subpopulation of patients with drug-resistant TB has not been replicated so far [69]. Taken together, serum CRP has outperformed symptom-based screening tools for active TB in people living with HIV, but its role in predicting treatment response seems to be limited [22].

This study has several strengths. We used various data sources to comprehensively explore factors potentially related to inflammation and address the raised scientific questions. The impact of other health conditions and natural variability on the study results was mitigated by applying exclusion criteria at the patient enrolment and, whenever required, considering common confounders as covariates in statistical analyses of the data.

There are also several study limitations. When conducting research in a low-endemic setting like Latvia (where the TB incidence in 2022 was 17 cases per 100,000 population, with a total population of 1.8 million [70]), one of the main shortcomings is the limited sample size, further narrowed by the exclusion criteria. Consequently, an unequal distribution of patients across multiple subcategories was observed. The impact of sample size was also evident in the variability of serum CRP levels, resulting in extremely positively skewed data distribution and thus requiring complex statistical approaches. Although the obtained results conform to the existing literature, they should be interpreted cautiously. Additionally, we did not record any cases of treatment failure or severe adverse events in our study population, precluding speculation on the role of inflammation. Lastly, another limitation is the lack of data on minimal inhibitory concentration (MIC), which would have allowed us to account for the susceptibility of the infecting Mtb isolates. Moreover, the combined PK/PD indices, such as C_max_/MIC and area under the time–concentration curve (AUC)/MIC, have been reported by others as useful in predicting treatment response and outcomes in patients with DS-TB [32,38,39].

## 4. Materials and Methods

### 4.1. Study Design and Population

From April 2017 to May 2023, we conducted a retrospective observational study of patients hospitalised in the Centre of Tuberculosis and Lung Diseases at Riga East University Hospital, who underwent the WHO-recommended treatment [2].

The diagnosis of DS-PTB was established based on the comprehensive initial clinical evaluation that included the following: (a) interpretation of clinical symptoms reported by the patient and the results of peripheral blood testing; (b) assessment of the radiological findings (chest X-ray supplemented with data from computer tomography in case of inconclusive findings); and (c) the data from bacteriological testing (sputum-smear microscopy [auramine–rhodamine staining], mycobacterial culture [Löwenstein–Jensen [LJ] media and mycobacteria growth incubator tubes [BACTEC MGIT 960 system, Becton Dickinson, Heidelberg, Germany]], and molecular assay [GeneXpert MTB/RIF Ultra, Cepheid, Sunnyvale, CA, USA]).

Patients were excluded from the study if the following criteria were applicable: (a) extrapulmonary TB; (b) culture-confirmed drug-resistant TB; (c) age < 18 years; (d) pregnancy or lactation; (e) severe acute or chronic conditions that may interfere with blood test results; (f) concomitant infectious diseases (e.g., HVB, HVC, and HIV); and (g) previous history of malignancy.

### 4.2. Clinical Data

The clinical information retrospectively retrieved from electronic medical records and patient questionnaires included the following: (a) serum CRP level determined prior to anti-TB treatment onset (CRP_b_) and, aligned with PK sampling, 10–12 days afterwards (CRP_10–12d_) to characterise the degree of inflammation at baseline and an early effect of therapy; (b) radiologically determined localisation of the TB-associated lung abnormalities such as consolidations and nodules (unilateral or bilateral lesions) and the presence or absence of the cavitations to determine the extent of lung involvement; (c) sputum-smear microscopy results (simplified to dichotomous variable—“positive” or “negative”) to distinguish infectious patients with a presumably higher bacillary load. In addition, the demographic and anthropometric information, comorbidities, concomitantly used drugs, and self-reported smoking status were also considered clinically relevant information. In conformity with WHO recommendations [44], a patient was classified as underweight if the BMI was <18.5 kg/m^2^ and overweight if the BMI was ≥25.0 kg/m^2^. The reference range for serum CRP levels, established by the clinical laboratory of the Centre of Tuberculosis and Lung Diseases at Riga East University Hospital, is <8 mg/L.

Since there are no clinical recommendations on the pattern or timeframe within which serum CRP levels should normalise in response to anti-TB treatment, we stratified patients for subgroup analyses based on a modified criterion proposed by Wilson et al. [20]. To further characterise the changes in the levels of this biomarker, patients were stratified into the following groups: Group A comprised patients whose serum CRP levels were within the reference range before starting anti-TB treatment and 10–12 days later; Group B—patients whose serum CRP levels reduced ≥2 times or reached the reference range; and Group C—patients whose serum CRP levels reduced <2 times, including cases when serum CRP levels remained unchanged or even increased.

### 4.3. Determination of the Anti-TB Drug Plasma Exposure Using LC-MS/MS

The measurements of anti-TB drug plasma concentration were performed when patients were in the intensive phase of treatment and routinely receiving ETB, PZA, RIF, and INH at daily doses of 12–25 mg/kg, 20–30 mg/kg, 8–12 mg/kg, and 4–6 mg/kg, respectively, as per the WHO guidelines [2]. PK sampling was performed at three consecutive time points—pre-dose (0 h), 2 and 6 h post-dose—between the 10th and 12th days of therapy, the earliest point at which anti-TB drug exposure could be assessed to ensure steady-state plasma concentration has been reached [71].

Venous blood was collected into BD Vacutainer tubes with EDTA coating (Becton Dickinson, Plymouth, UK) and promptly centrifuged at 4000 rpm (3488 g) at +4 °C for 15 min to separate and harvest the plasma. The obtained samples were stored frozen at −70 °C until analysis. Then, plasma samples were processed and analysed adhering to the LC-MS/MS protocol described earlier [72]. The patients were classified as underexposed if the plasma concentration of the respective anti-TB drug 2 h post-dose (at C_max_) was below the expected range: 2–6 µg/mL for ETB, 20–60 µg/mL for PZA, 8–24 µg/mL for RIF, and 3–6 µg/mL for INH [45]. The total plasma exposure was evaluated using the AUC from 0 to 6 h (AUC_0–6h_) constructed from the data over the three time points mentioned above. Missing values, if any, were replaced with values corresponding to ½ of the lower limit of quantification of the respective drug [72].

### 4.4. Assessment of Treatment Response Employing a Culture-Based Approach

The response to anti-TB treatment was assessed using the results from sputum culture on LJ media, aimed at detecting viable Mtb. The testing frequency depended on the sputum-smear status at the baseline—once a month for smear-negative patients and twice a month for smear-positive patients. The primary endpoint was tSCC, and it was defined as the number of days from the start of anti-TB treatment to the sample collection date (including) of the first from two sputum cultures consecutively reported to be negative. Cases were censored to the last available sample collection date if a patient was lost to follow-up or completed treatment but whose treatment outcome did not comply with the WHO definition of cure [11].

### 4.5. Statistical Data Analysis

All the quantitative variables were assessed for data conformity to a normal distribution by performing the Shapiro–Wilk test and visually inspecting data frequency distribution histograms. Whenever required, non-normally distributed variables (BMI, tSCC, ETB dose and C_max_, RIF C_max_ and AUC_0–6h_, INH AUC_0–6h_, CRP_b_, and CRP_10–12d_) were log-transformed before the analyses to reduce data skewness and conform statistical assumptions. Qualitative variables were summarised using counts and percentages and analysed using the Chi-square or Fisher’s exact test, where applicable. Quantitative variables were given as the mean and standard deviation (±SD) for normally distributed data and the median with interquartile range (IQR) for non-normally distributed data; group comparisons were performed using Student’s *t*-test or one-way ANOVA, along with their non-parametric equivalents as the Mann–Whitney U test or the Kruskal–Wallis H test for non-normally distributed data. Additionally, Spearman’s rank correlation was required for the correlation analysis of non-normally distributed variables. The CRP_b_ and CRP_10–12d_ levels were compared using the Wilcoxon signed-rank test. Linear regression was applied to examine the relationship between CRP_10–12d_ levels and PK parameters of each anti-TB drug, adjusted for sex, age, and BMI. Next, ANCOVA was conducted to compare the anti-TB drug exposure in the groups based on the fold change in the CRP levels over the specified time, considering biological sex, age, and drug dose as covariates.

In the end, time-to-event analysis incorporating Kaplan–Meier curves and Cox proportional hazards regression were employed to study the association between the serum CRP level reduction and tSCC.

The statistical data analysis was performed using the software IBM SPSS Statistics (version 29.0.0.0, IBM Corp: Armonk, NY, USA) and R (version 4.2.1, R Core Team (2022): Vienna, Austria, R Foundation for Statistical Computing, https://r-project.org, accessed on 10 September 2024). The statistically significant findings were indicated by a two-sided *p* value of less than 0.05.

During the preparation of this manuscript, AI-based tools ChatGPT 3.5 (version October 2023, OpenAI, https://chat.openai.com/chat, accessed on 10 September 2024) and Grammarly (version 14.1086.0, Grammarly, https://app.grammarly.com/, accessed on 10 September 2024) were used for English language editing.

## 5. Conclusions

Summarising all the above, we provided novel insights into TB-associated inflammation by identifying patient characteristics and findings from the initial clinical evaluation, which were related to delayed reduction in serum CRP levels shortly after anti-TB treatment onset. In the present setting, the variability in anti-TB drug plasma exposure did not directly impact the early changes in the levels of this biomarker. Furthermore, the serum CRP levels measured after 10–12 days of anti-TB drug administration, or the fold change from the baseline, did not predict the treatment response. Thus, in our view, the assessment of the serum CRP levels at this time point lacks clinical relevance, at least in patients without comorbidities.

## Figures and Tables

**Table 1:** Characteristics of the study population (*N* = 42).

Demographic, anthropometric, and lifestyle characteristics		
Biological sex	Male, *n* (%)	34 (81.0)
	Female, *n* (%)	8 (19.0)
Age	Overall, years (mean [±SD])	47 (±14)
	<60 years, *n* (%)	34 (81.0)
	≥60 years, *n* (%)	8 (18.0)
BMI	Overall, kg/m^2^ (median [IQR]) ^a^	20.9 (18.4–22.7)
	Underweight, *n* (%)	11 (26.2)
	Normal weight, *n* (%)	25 (59.5)
	Overweight, *n* (%)	6 (14.3)
Smoking status	Smoker, *n* (%)	31 (73.8)
	Non-smoker, *n* (%)	11 (26.2)
Baseline radiological findings		
Localisation of lung lesions	Unilateral, *n* (%)	11 (26.2)
	Bilateral, *n* (%)	31 (73.8)
Cavitations	Present, *n* (%)	26 (61.9)
	Absent, *n* (%)	16 (38.1)
Baseline sputum-smear microscopy results		
	Positive, *n* (%)	27 (64.3)
	Negative, *n* (%)	15 (35.7)
Bacteriological response to treatment		
tSCC	Overall, days (median [IQR])	56 (27–79)

Qualitative variables are expressed as counts (percentage). The quantitative, normally distributed variables are presented as mean and standard deviation (±SD), while the non-normally distributed variables are expressed as median and interquartile range (IQR). ^a^ In conformity with WHO recommendations [44], a patient was classified as underweight if the BMI was <18.5 kg/m^2^ and overweight if the BMI was ≥25.0 kg/m^2^. Abbreviations: BMI—body mass index; tSCC—time to sputum culture conversion.

**Table 2:** Distribution of serum CRP levels in the study population and comparison within subcategories.

	CRP_b_, mg/L ^a^	*p* Value	CRP_10–12d_, mg/L ^a,b^	*p* Value
Overall	21.9 (3.3–51.5)	N/A	6.4 (1.4–34.2)	N/A
Demographic, anthropometric, and lifestyle characteristics				
Biological sex				
Male	21.9 (5.4–56.2)	0.421	6.8 (2.6–34.2)	0.235
Female	15.0 (1.4–47.7)		1.5 (0.9–42.4)	
Age, years	–	0.882	–	0.161
<60 years	21.9 (3.1–56.2)	0.718	4.8 (1.4–24.8)	0.248
≥60 years	21.8 (9.3–47.7)		18.0 (3.2–64.7)	
BMI, kg/m^2 c^	–	0.011	–	0.035
Underweight	55.5 (24.4–79.0)	0.040	34.2 (10.9–65.3)	0.116
Normal weight	16.0 (3.3–41.4)		4.0 (1.4–19.5)	
Overweight	5.2 (1.3–24.4)		4.1 (1.1–15.7)	
Smoking status				
Smoker	22.8 (5.7–58.3)	0.163	12.1 (3.2–45.0)	0.046
Non-smoker	8.2 (1.1–32.2)		1.8 (0.8–14.1)	
Radiological findings				
Localisation of lung lesions				
Unilateral	19.1 (1.1–40.3)	0.365	2.2 (1.2–14.2)	0.249
Bilateral	24.4 (4.4–55.5)		6.6 (2.7–48.5)	
Cavitations				
Present	36.3 (19.8–66.6)	<0.001	14.2 (6.2–60.9)	<0.001
Absent	3.0 (1.1–7.6)		1.4 (0.7–3.2)	
Baseline sputum-smear microscopy results				
Positive	29.2 (8.2–60.6)	0.011	13.5 (4.0–52.0)	<0.001
Negative	3.4 (1.1–26.6)		1.3 (0.6–6.2)	

All variables are expressed as median and interquartile range (IQR). The groups were compared using the Mann–Whitney U test or Kruskall–Wallis H test. The correlation analysis was performed using Spearman’s rank correlation. A *p* value of <0.05 was considered statistically significant. ^a^ The serum CRP reference range established by the clinical laboratory of the Centre of Tuberculosis and Lung Diseases at Riga East University Hospital is <8 mg/L. ^b^ Data were available for 41 patients. ^c^ In conformity with WHO recommendations [44], a patient was classified as underweight if the BMI was <18.5 kg/m^2^ and overweight if the BMI was ≥25.0 kg/m^2^. Abbreviations: CRP—C-reactive protein; CRP_b_—serum C-reactive protein level at the baseline; CRP_10–12d—_serum C-reactive protein level 10–12 days after anti-tuberculosis treatment onset; BMI—body mass index; N/A—not applicable.

**Table 3:** Assessment of anti-TB drug plasma exposure and relationship with serum CRP levels determined 10–12 days after treatment onset.

	Anti-TB Drug Doses and Pharmacokinetic Parameters ^a^		Linear Regression—Relationship with Serum CRP_10–12d_ Levels ^c^		
		Mean (±SD)	β	95% CI	*p* value
RIF	Dose, mg/kg	9.5 (±1.5)	–	–	–
	C_max_, µg/mL	1.89 (0.32–5.50) ^b^	−0.012	−0.069, 0.064	0.934
	AUC_0–6h_, µg × h/mL	12.82 (6.75–22.54) ^b^	0.092	−0.012, 0.024	0.520
PZA	Dose, mg/kg	31.2 (±4.7)	–	–	–
	C_max_, µg/mL	36.74 (±11.85)	−0.074	−0.023, 0.013	0.597
	AUC_0–6h_, µg × h/mL	187.18 (±49.65)	−0.094	−0.006, 0.003	0.505
ETB	Dose, mg/kg	21.0 (18.1–23.2) ^b^	–	–	–
	C_max_, µg/mL	2.22 (1.48–3.91) ^b^	−0.155	−0.216, 0.065	0.282
	AUC_0–6h_, µg × h/mL	13.00 (±4.97)	−0.148	−0.065, 0.020	0.295
INH	Dose, mg/kg	4.8 (±0.8)	–	–	–
	C_max_, µg/mL	2.64 (±1.38)	0.128	−0.086, 0.227	0.365
	AUC_0–6h_, µg × h/mL	10.02 (6.38–14.58) ^b^	0.140	−0.019, 0.057	0.321

The relationship between PK parameters and log-transformed CRP_10–12d_ levels was tested using linear regression adjusted for the patient’s biological sex, age, and BMI. A *p* value of <0.05 was considered statistically significant. ^a^ Data were available for 42 patients. ^b^ The variable is presented as the median and interquartile range (IQR). ^c^ Data were available for 41 patients. Abbreviations: TB—tuberculosis; CRP_10–12d—_serum C-reactive protein level 10–12 days after anti-tuberculosis treatment onset; SD—standard deviation; β—standardised beta coefficient; CI—confidence interval; RIF—rifampicin; PZA—pyrazinamide; ETB—ethambutol; INH—isoniazid; C_max—_peak plasma concentration measured 2 h post-dose; AUC_0–6h_—area under the time–concentration curve from 0 to 6 h post-dose.

**Table 4:** Multivariate Cox proportional hazard models of patient characteristics predicting time to sputum culture conversion.

	Model 1		Model 2		Model 3		Model 4		Model 5	
Predictors	HR (95% CI)	*p* Value	HR (95% CI)	*p* Value	HR (95% CI)	*p* Value	HR (95% CI)	*p* Value	HR (95% CI)	*p* Value
Male sex									0.82 (0.29–2.30)	0.696
Age, years					1.00 (0.66–1.50)	0.947				
≥60 years	0.94 (0.37–2.40)	0.902	0.92 (0.36–2.40)	0.862						
BMI_,_ kg/m^2^	1.39 (0.91–2.10)	0.130	1.35 (0.90–2.00)	0.153	1.30 (0.84–2.00)	0.248	1.53 (0.99–2.40)	0.056	1.43 (0.97–2.10)	0.072
Smoker	1.58 (0.69–3.60)	0.276					1.83 (0.73–4.60)	0.194	1.82 (0.69–4.80)	0.228
Lung cavitations			1.35 (0.60–3.00)	0.465						
Positive baseline sputum-smear microscopy results							0.75 (0.29–1.90)	0.556		
RIF AUC_0–6h_					1.30 (0.95–1.80)	0.105				
CRP_10–12d_ level	0.96 (0.69–1.30)	0.792	0.93 (0.64–1.40)	0.704	1.00 (0.73–1.50)	0.883				
Serum CRP level changes from the baseline										
Group A							1.37 (0.56–3.40)	0.497	1.37 (0.55–3.40)	0.498
Group B							Reference	N/A	Reference	N/A
Group C							1.65 (0.66–4.10)	0.285	1.49 (0.65–3.40)	0.350
Global *p* value		0.444		0.556		0.307		0.425		0.448
Concordance index		0.56		0.57		0.63		0.56		0.55

All continuous predictors were scaled before inclusion in the regression model. Group A—serum CRP levels were within the reference range at both time points; Group B—serum CRP levels decreased by ≥2 times from the first to the second time point or reached the reference range at the second time point; Group C—serum CRP levels decreased by <2 times from the first to second time point. A *p* value of <0.05 was considered statistically significant. Abbreviations: TB—tuberculosis; RIF—rifampicin; AUC_0–6h_—area under the time–concentration curve from 0 to 6 h post-dose, CRP—C-reactive protein; CRP_10–12d_—serum C-reactive protein level 10–12 days after anti-tuberculosis treatment onset; HR—hazard ratio; CI—confidence interval; N/A—not applicable.

## Data Availability

The datasets supporting the findings of this study are not publicly available due to ethical restrictions but are available from the corresponding author upon reasonable request.

## Supplementary Materials

The following supporting information can be downloaded at: https://www.mdpi.com/article/10.3390/antibiotics13121216/s1, Table S1: Comparison of patient characteristics after stratification based on changes in serum CRP levels 10–12 days after treatment onset; Table S2: Comparison of anti-TB drug plasma exposure after patient stratification based on changes in serum CRP levels 10–12 days after anti-tuberculosis treatment onset; Table S3: Univariate Cox proportional hazard models of patient characteristics predicting time to sputum culture conversion.
