# Supplementary material for: Identification of Factors Determining Patterns of Serum C-Reactive Protein Level Reduction in Response to Treatment Initiation in Patients with Drug-Susceptible Pulmonary Tuberculosis
Source: Antibiotics (Basel). 2024 Dec 14;13(12):1216. doi: 10.3390/antibiotics13121216 (PMC11672611; doi:10.3390/antibiotics13121216)
Supplement: Supplementary file 1 [file antibiotics-13-01216-s001.zip › antibiotics-3289777-supplementary.pdf]

Table S1 Comparison of patient characteristics after stratification based on changes in serum CRP levels 10–12 days after treatment onset

	Group A (n = 13)	Group B (n = 14)	Group C (n = 14)	p value
Demographic, anthropometric and lifestyle characteristics				
Biological sex				
Male, n (%)	9 (69.2)	12 (85.7)	12 (85.7)	0.545
Female, n (%)	4 (30.8)	2 (14.3)	2 (14.3)	
Age, years (mean [± SD])	43 (± 13)	47 (± 15)	52 (± 13)	0.293
< 60 years, n (%)	12 (92.3)	11 (78.6)	10 (71.4)	0.483
≥ 60 years, n (%)	1 (7.7)	3 (21.4)	4 (28.6)	
BMI, kg/m^2 (median [IQR]) [a]	22.3 (20.3–25.0)	21.1 (18.2–22.7)	19.0 (17.9–21.5)	0.074
Underweight, n (%)	1 (7.7)	4 (28.6)	5 (35.7)	0.464
Normal weight, n (%)	9 (69.2)	8 (57.1)	8 (57.1)	
Overweight, n (%)	3 (23.1)	2 (14.3)	1 (7.1)	
Smoking status				
Smoker, n (%)	8 (61.5)	10 (71.4)	12 (85.7)	0.377
Non-smoker, n (%)	5 (38.5)	4 (28.6)	2 (14.3)	
Radiological findings				
Localisation of lung lesions				
Unilateral, n (%)	4 (30.8)	4 (28.6)	3 (21.4)	0.909
Bilateral, n (%)	9 (69.2)	10 (71.4)	11 (78.6)	
Cavitations				
Present, n (%)	2 (15.4)	11 (78.6)	12 (85.7)	< 0.001
Absent, n (%)	11 (84.6)	3 (21.4)	2 (14.3)	
Baseline sputum-smear microscopy result				
Positive, n (%)	5 (38.5)	9 (64.3)	13 (92.9)	0.010
Negative, n (%)	8 (61.5)	6 (35.7)	1 (7.1)	
Bacteriological response to treatment				
tSCC, days (median [IQR])	46 (27–77)	46 (25–73)	66 (36–86)	0.626

Qualitative variables are expressed as counts (percentage), and groups were compared using the Chi-square or Fisher's exact test. The quantitative, normally distributed variables are presented as mean and standard deviation (± SD) and analysed using the one-way ANOVA. The quantitative, non-normally distributed variables are given as median and interquartile range (IQR) and analysed using the Kruskal-Wallis H test. For all tests, a p value of < 0.05 was considered statistically significant.

Group A – serum CRP levels were within the reference range at both time points; Group B – serum CRP levels decreased by ≥ 2 times from the first to the second time point or reached the reference range at the second time point; Group C – serum CRP levels decreased by < 2 times from the first to second time point.

[a] In conformity with WHO recommendations [1], a patient was classified as underweight if the BMI was < 18.5 kg/m^2 and overweight if the BMI was ≥ 25.0 kg/m^2.

Abbreviations: BMI – body mass index; tSCC – time to sputum culture conversion.

Table S2 Comparison of anti-TB drug plasma exposure after patient stratification based on changes in serum CRP levels 10–12 days after anti-tuberculosis treatment onset

	Group A (*n* = 13)	Group B (*n* = 14)	Group C (*n* = 14)	*p* value
RIF				
C_{max}, µg/mL	1.40 (± 1.54)	1.33 (± 1.46)	1.47 (± 1.52)	0.985 [a]
$AUC_{0–6h}$, µg × h/mL	10.09 (± 1.26)	12.11 (± 1.23)	14.39 (± 1.25)	0.611
PZA				
C_{max}, µg/mL	38.36 (± 3.78)	37.77 (± 3.34)	33.72 (± 3.65)	0.657
$AUC_{0–6h}$, µg × h/mL	194.91 (± 15.61)	192.88 (± 13.81)	173.78 (± 15.09)	0.590
ETB				
C_{max}, µg/mL	2.90 (± 1.22)	2.26 (± 1.20)	1.72 (± 1.22)	0.236 [b]
$AUC_{0–6h}$, µg × h/mL	14.44 (± 1.53)	13.19 (± 1.40)	11.43 (± 1.52)	0.430
INH				
C_{max}, µg/mL	2.19 (± 0.44)	3.01 (± 0.38)	2.73 (± 0.42)	0.371
$AUC_{0–6h}$, µg × h/mL	8.39 (± 1.19)	11.69 (± 1.16)	9.25 (± 1.18)	0.294 [c]

The pharmacokinetic parameters were compared across groups using the ANCOVA after controlling for confounders (biological sex, age, and drug dose) and described using estimated marginal mean (± standard error); a *p* value of < 0.05 was considered statistically significant.

[a] The result should be interpreted with caution. Although RIF C_{max} was log-transformed before inclusion in the model, data skewness was not entirely eliminated.

[b] ETB C_{max} was log-transformed before inclusion in the model.

[c] IZN $AUC_{0–6h}$ was log-transformed before inclusion in the model.

Group A – serum CRP levels were within the reference range at both time points; Group B – serum CRP levels decreased by ≥ 2 times from the first to the second time point or reached the reference range at the second time point; Group C – serum CRP levels decreased by < 2 times from the first to second time point.

Abbreviations: RIF – rifampicin; PZA – pyrazinamide; ETB – ethambutol; INH – isoniazid; C_{max} – peak plasma concentration measured 2 hours post-dose; $AUC_{0–6h}$ – area under the time-concentration curve from 0 to 6 hours post-dose.

Table S3 Univariate Cox proportional hazard models of patient characteristics predicting time to sputum culture conversion

Predictors	HR (95% CI)	*p* value
Demographic, anthropometric and lifestyle characteristics		
Biological sex		
Male	0.95 (0.42, 2.17)	0.903
Female	Reference	N/A
Age, years	1.19 (0.85, 1.68)	0.309
< 60 years	Reference	N/A
≥ 60 years	1.30 (0.58, 2.80)	0.546
BMI, kg/m^2 [a]	1.28 (0.93, 1.76)	0.125
Underweight	0.55 (0.24, 1.20)	0.148
Normal weight	Reference	N/A
Overweight	1.50 (0.61, 3.70)	0.376
Smoking status		
Smoker	1.29 (0.60, 2.76)	0.515
Non-smoker	Reference	N/A
Radiological findings		
Localisation of lung lesions		
Unilateral	Reference	N/A
Bilateral	1.10 (0.50, 2.20)	0.890
Cavitations		
Present	1.20 (0.59, 2.46)	0.617
Absent	Reference	N/A
Baseline sputum-smear microscopy result		
Positive	1.12 (0.57, 2.19)	0.751
Negative	Reference	N/A
Anti-TB drug exposure		
RIF $AUC_{0–6h}$	1.30 (0.95, 1.77)	0.102
PZA $AUC_{0–6h}$	1.03 (0.74, 1.42)	0.872
ETB $AUC_{0–6h}$	1.23 (0.90, 1.68)	0.193
INH $AUC_{0–6h}$	0.93 (0.64, 1.34)	0.695
Serum CRP levels		
CRP_b level	0.96 (0.67, 1.38)	0.832
$CRP_{10–12d}$ level	0.93 (0.69, 1.28)	0.669
Changes from baseline		
Group A	1.29 (0.56, 3.00)	0.551
Group B	Reference	N/A
Group C	1.26 (0.57, 2.80)	0.571

A *p* value of < 0.05 was considered statistically significant.

[a] In conformity with WHO recommendations [1], a patient was classified as underweight if the BMI was < 18.5 kg/m^2 and overweight if the BMI was ≥ 25.0 kg/m^2.

Group A – serum CRP levels were within the reference range at both time points; Group B – serum CRP levels decreased by ≥ 2 times from the first to the second time point or reached the reference range at the second time point; Group C – serum CRP levels decreased by < 2 times from the first to second time point.

Abbreviations: BMI – body mass index; TB – tuberculosis; RIF – rifampicin; PZA – pyrazinamide; ETB – ethambutol; INH – isoniazid; C_{max} – peak plasma concentration measured 2 hours post-dose; $AUC_{0–6h}$ – area under the time-concentration curve from 0 to 6 hours post-dose, CRP – C-reactive protein; CRP_b – serum C-reactive protein level at the baseline; $CRP_{10–12d}$ – serum C-reactive protein level 10–12 days after anti-tuberculosis treatment onset; HR – hazard ratio; CI – confidence interval; N/A – not applicable.

References

1. World Health Organization. *A Healthy Lifestyle–WHO Recommendations*; World Health Organization: Geneva, Switzerland, 2010. Available online: https://www.who.int/europe/news-room/fact-sheets/item/a-healthy-lifestyle---who-recommendations (accessed on 8 May 2024).
